# Postgenomic Approaches and Bioinformatics Tools to Advance the Development of Vaccines against Bacteria of the *Burkholderia cepacia* Complex

**DOI:** 10.3390/vaccines6020034

**Published:** 2018-06-08

**Authors:** Sílvia A. Sousa, António M. M. Seixas, Jorge H. Leitão

**Affiliations:** IBB—Institute for Biotechnology and Biosciences, Department of Bioengineering, Instituto Superior Técnico, Universidade de Lisboa, 1049-001 Lisboa, Portugal; sousasilvia@tecnico.ulisboa.pt (S.A.S.); antonio.seixas@tecnico.ulisboa.pt (A.M.M.S.)

**Keywords:** Bcc vaccines, immunoproteomics, epitope prediction web tools

## Abstract

Bacteria of the *Burkholderia cepacia* complex (Bcc) remain an important cause of morbidity and mortality among patients suffering from cystic fibrosis. Eradication of these pathogens by antimicrobial therapy often fails, highlighting the need to develop novel strategies to eradicate infections. Vaccines are attractive since they can confer protection to particularly vulnerable patients, as is the case of cystic fibrosis patients. Several studies have identified specific virulence factors and proteins as potential subunit vaccine candidates. So far, no vaccine is available to protect from Bcc infections. In the present work, we review the most promising postgenomic approaches and selected web tools available to speed up the identification of immunogenic proteins with the potential of conferring protection against Bcc infections.

## 1. Introduction

*Burkholderia cepacia* was initially described in 1950 by Walter Burkholder as the phytopathogen formerly named *Pseudomonas cepacia* [1]. This organism emerged in the 1980s as an important opportunistic pathogen of patients suffering from cystic fibrosis (CF). Since then, several outbreaks were reported in several CF care units in Europe and North America, causing premature deaths among these patients [2]. Although *B. cepacia* remains as the type species, the remarkable advances achieved in the molecular taxonomy of the bacterium led to the recognition that it is in fact not a single species, but several closely related species that infect CF patients. Together, these bacterial species comprise the *Burkholderia cepacia* complex (Bcc), which contains at least 24 distinct species [3,4,5,6]. Although virtually all Bcc species are capable of causing severe and life-threatening infections to these already debilitated patients, the Bcc species *B. cenocepacia* and *B. multivorans* account for the vast majority of infections among CF patients worldwide [7,8]. Despite regional variations, the incidence of Bcc infections among CF patients is presently lower than 5% [9,10,11], although in the late 1990s, the incidence was much higher, with several CF centers experiencing outbreaks with an incidence rate as high as 20% [12]. Much of the research work on these bacteria stems from the unpredictable outcome of the infections, which upon colonization, ranges from asymptomatic carriage to the development of cepacia syndrome, a fatal pneumonia accompanied by fever and septicemia that can develop within a period of time as short as a week [13]. The unpredictability of the infection outcome, combined with the intrinsic and acquired resistance to antibiotics and the rapid patient-to-patient spread of epidemic strains, rendered these infections particularly feared by patients and caregivers. Infection by Bcc is reported to lead to a 2.5-fold reduction of CF patients’ life expectancy [14,15]. In recent years, Bcc also emerged as important pathogens among non-CF patients, in particular patients hospitalized for prolonged periods and suffering from malignancies such as cancer [16]. 

The eradication of Bcc infections remains a challenge. Therapies with antibiotics provide limited success, and their effectiveness among chronically infected patients with Bcc often fails. The only successful strategies to reduce the incidence of Bcc infections were the segregation measures took by several CF centers experiencing outbreaks, with a heavy psychosocial burden to the patients [17]. Therefore, novel strategies to protect patients from Bcc infections are urgently needed. In this context, vaccines that can confer protection against Bcc infections are a promising and attractive strategy, as they have proven to be highly effective in preventing infections caused by various bacterial pathogens [18]. Since no vaccines are clinically available to prevent Bcc infection, there is a critical need to rapidly develop vaccine candidates. In this work, we review the powerful tools presently available, resulting from postgenomic knowledge and bioinformatic web tools, to design and develop protective vaccines.

## 2. Which Is the Best Type of Vaccine for Bcc?

No vaccines are currently available to protect against Bcc infections. In general, vaccines against other bacterial human pathogens can be grouped into different types such as live attenuated vaccines, killed whole-cell vaccines, toxoid vaccines, or subunit vaccines. Killed whole-cell vaccines are in use to prevent anthrax, Q fever, and whooping cough [19]. Killed whole-cell vaccines are known for inducing narrow immune responses resulting from the inability of the pathogen to replicate within the host, and do not confer cellular immunity, an important feature for intracellular pathogens such as Bcc. Several studies using killed whole cells of the Bcc-related *B. pseudomallei* and *B. mallei* as vaccines in mice showed that these vaccines were ineffective in conferring protection against subsequent challenges (reviewed by [20]). No reports on the use of this type of vaccines against Bcc are available. Live attenuated vaccines are able to replicate within the host, but do not cause disease, being at the same time able to stimulate both a humoral and a cellular immune response. Different strains and vaccination protocols have been described for *B. pseudomallei* and *B. mallei* using mice as infection models (reviewed by [20]). Pradenas and colleagues have very recently reported the use of a *B. cenocepacia* strain with a mutation in the *tonB* gene as a potential live attenuated vaccine that protected against acute respiratory infection in BALB/c mice [21]. No other examples can be found in the literature on the use of live attenuated or killed whole-cell vaccines to combat infections by Bcc. Contrastingly, several virulence factors and proteins have been identified for future use as subunit vaccines [22,23,24,25,26,27]. Surface and extracytoplasmatic molecules/structures of pathogens are the first exposed to the host immune system, and therefore expected to trigger the host immune response. These surface molecules/structures are mainly composed of proteins and polysaccharides. Polysaccharide-conjugated vaccines are already in use, such as the pneumococcal vaccine and conjugated vaccines to prevent meningococcal disease. Bcc are known to produce various extracellular polysaccharides, including cepacian and others [28,29,30,31]. Several roles related to stress resistance and protection of the pathogen from the immune system have been attributed to cepacian, but no studies on the possible use as a vaccine has been reported so far. The limited information on the use of complex carbohydrates or polysaccharides as components of vaccines against Bcc infections was recently reviewed by Pradenas et al. [32]. Therefore, polysaccharide-based vaccines against Bcc will not be addressed in this work.

Proteins, and especially surface-exposed proteins, appear as the most attractive group of molecules to exploit for the future development of subunit vaccines [33]. Cytoplasmic proteins of the pathogen should also be considered, since some of these proteins, known as “moonlighting proteins”, can also appear at the bacterial surface and be immunoreactive and immunoprotective [34,35]. In addition, proteins are the class of molecules for which a wide range of bioinformatics tools and experimental methodologies are better developed. In the next sections, some of the experimental and bioinformatics approaches in use to aid in the task of identifying immunoreactive proteins of potential use as subunit vaccines are presented and discussed.

## 3. Experimental Techniques to Identify Immunogenic Proteins

Developments in genomics, proteomics, and bioinformatics can accelerate the discovery of antigens from Bcc. Several techniques combining information from these fast-developing areas can therefore be used to unveil potential immunogenic proteins. Many of these techniques rely on the use of proteomics, and include serological proteome analysis, high-throughput proteome arrays, glycopetide arrays, surface shaving approaches, preadsorbed immunoproteomics, selective biotinylation approaches, and immunocapture mass spectrometry [22,36,37,38,39,40,41].

**Figure 1:**
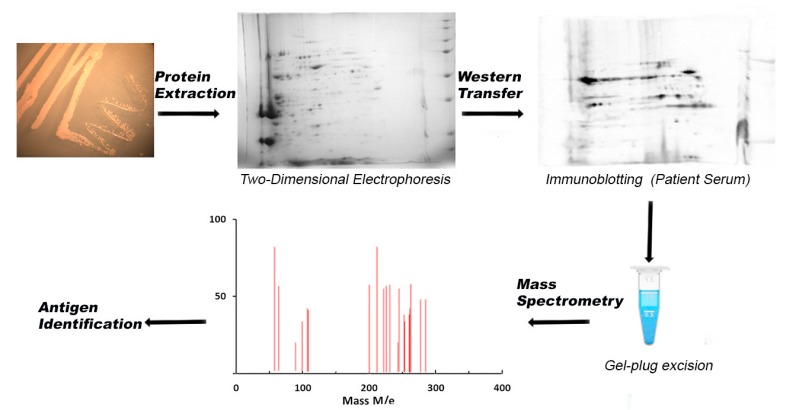
Schematic flowchart of the steps involved in the identification of a pathogen immunoproteome, illustrating bacterial protein extraction, protein separation by 2D gels, Western blotting using patient serum samples, and protein identification by mass spectrometry.

Immunoproteome analysis is one of the most common proteomics-based techniques used to identify antigens of potential interest for vaccine development. This technique relies on the fractionation of protein samples by 2D-PAGE according to their isoelectric point (pI) and molecular mass (MW). After this fractionation, Western blot procedures are used to reveal the immunoreactive proteins. Immunoreactive proteins are then excised from the gel, processed, and identified by mass spectrometry techniques (Figure 1).

Although the methodology is quite popular, it presents several technical difficulties. A major difficulty is the solubilization of proteins, particularly proteins that are hydrophobic or membrane-associated, as well as highly acidic or basic, or proteins with a low or a high molecular mass. Low-abundance proteins are also difficult to identify by mass spectrometry. In 2013, Shinoy and colleagues used a serological proteomics approach to identify immunogenic proteins from *B. cenocepacia* and *B. multivorans*, the two most prevalent Bcc species worldwide [22]. These authors performed a 2D-PAGE followed by a Western blot with serum from CF patients with a record of infection by Bcc. A total of 15 common immunoreactive bacterial proteins were found. Although further studies are required, these immunogenic proteins are potential candidates to develop vaccines to prevent Bcc infections. Our research group has recently used a similar approach to identify immunogenic proteins from cells of the *B. cenocepacia* J2315 strain cultivated on an artificial sputum medium at 37 °C in aerobic or microaerophilic conditions, mimicking the CF lung environment. Nineteen proteins were found to be immunoreactive to serum samples from Bcc-infected CF patients, and five extracytoplasmic proteins were found to be highly conserved among Bcc members. 

A variant technique, named surface shaving, was developed by Olaya-Abril and coworkers [36]. The methodology was designed to specifically recover surface-exposed proteins and to avoid the problems associated with 2D-PAGE fractionation of membrane proteins by analyzing the “shaved” surface proteins directly by liquid chromatography coupled to mass spectrometry (LC/MS/MS). Surface-exposed proteins play critical roles in the interaction between the cell and the surrounding environment, contributing to the bacterial physiology and pathogenesis and including transport, adhesion, and signaling functions, as well as roles in virulence and cytotoxicity processes. Surface-exposed proteins are involved in the initial interactions with the host cell and have higher probability of being immunoreactive. In the proposed methodology, living bacterial cells (or other type of cells) are subjected to protease digestion in conditions avoiding cell lysis, so that the exposed moieties of surface proteins can be recovered by centrifugation and filtration. The purified and digested surface proteins are then identified by LC/MS/MS. In this methodology, live bacteria are incubated with proteases to “shave” surface-exposed moieties, while the usually-followed protocol in immunoproteomics includes cell lysis followed by centrifugation to obtain membrane and cytoplasmic proteins. The methodology, with variants, has been applied to several bacterial pathogens. Unexpectedly, the recovery of cytoplasmic proteins has been reported when using “shaving” procedures [36]. The occurrence of these cytoplasmic proteins has been hypothesized to result from cell lysis during sample processing, from their exportation by noncanonical secretion systems, or due to their release by outer membrane vesicles [36].

Conventional serological techniques often use cross-absorption procedures to avoid cross reactions during agglutination assays [42]. Cross-absorption techniques have been used to improve the accuracy of agglutination diagnoses, originating a novel “preadsorbed” serum lacking the antibodies that recognized surface antigens of the bacterial pathogen. The preadsorbed immunoproteomic approach combines the use of proteomics techniques with the use of preadsorbed sera. In this methodology, proteins from the pathogen are processed and separated by 2D-PAGE procedures, followed by Western blot using the normal serum and the preadsorbed serum. Protein spots appearing when using normal serum, but not when the preadsorbed serum is used, are predicted to be surface antigens. The identified spots can then be excised from gels and proteins can be identified by MALDI-TOF MS. This methodology was used to identify surface proteins of *Streptococcus suis* [37]. The proteins identified were later confirmed to be located at the bacterial cell surface by bioinformatic predictions and immunofluorescence assays [37].

Methodologies based on the labeling of surface proteins by chemical modifications have also been successfully developed. This includes the case of biotinylation, initially used for eukaryotic cells [43] and then extended to both Gram-positive and Gram-negative bacteria [38,44]. The methodology involves the labeling of surface proteins with biotin, cell disruption and processing, enrichment by avidin-affinity chromatography, 2D-PAGE separation, and identification by techniques such as MALDI-TOF MS. The methodology was used with success for the pathogens *H. pylori* and *B. pseudomallei* [38,39]. In the case of *B. pseudomallei*, Harding et al. [38] identified a total of 35 surface proteins using a biotinylation approach and 12 of them were confirmed as immunoreactive using sera from infected patients.

Immunocapture is another methodology that can be used to identify immunogenic proteins from a pathogen. In this methodology, immunoglobulins from a patient with a record of infection are immobilized in protein A or protein G. The process usually uses immunoglobulins immobilized in a column where the protein samples from a pathogen are introduced. The pathogen proteins captured are then recovered and analyzed by mass spectrometry techniques [40]. Several variants to this technique have been described, mainly to identify cancer-related auto-antigens [45]. 

Protein arrays are a promising technology to identify immunogenic proteins, with the advantage that virtually all proteins from a pathogen can be spotted on a microarray, surpassing the limitation of the low abundance of some immunoreactive proteins. This limitation has been reported, for example, by Shinoy et al., who found several immunoreactive proteins, but failed in their identification due to low abundance [22]. Our research group also found a similar problem when using immunoproteomics approaches to identify proteins of *B. cenocepacia* J2315 that react with serum from CF patients with a clinical record of Bcc infection. The protein array technique is based on the knowledge of complete bacterial genomes, and each open reading frame (ORF) of interest can be amplified by PCR techniques and the gene cloned in adequate expression vectors. The ORFs of interest can be selected based on bioinformatics tools (see Section 4) or experimental evidence of their immunoreactivity. After purification, proteins are imprinted in a microarray. The microarrays can be probed with serum samples and the immunoreactivity of proteins can be detected by using secondary antibodies conjugated with a fluorescent dye. Several advantages can be pointed out for this technique, including the possibility of analyzing the whole genome, use of nearly equal amounts of each protein despite their relative expression by the pathogen, and reduced amounts of serum and other chemicals required for screenings. These advantages, combined with the high throughput of microarray processing and analysis that allows the scanning of large numbers of serum samples in a short time, makes protein microarrays the ideal method by which to scan hundreds of serum samples. However, protein cloning and purification are potential bottlenecks, as it is still challenging to efficiently overexpress and purify highly hydrophobic proteins. In addition, post-translational modifications and native folding are often determinant features of immunogenicity that can be lost when using this methodology. Despite these limitations, Suwannasaen and colleagues screened protein microarrays containing 154 *B. pseudomallei* proteins with sera from 108 healthy individuals and 72 patients with a record of *B. pseudomallei* infections [41]. These authors were able to find novel immunoreactive proteins from *B. pseudomallei* which are potential candidates for development of subunit-based vaccines.

## 4. Bioinformatics Tools to Predict Immunogenic Epitopes

The genomic era brought new approaches for the design of vaccines, without the need to grow the pathogens. One of the approaches was “reverse vaccinology”, which relies on the whole genome screening using bioinformatics tools to identify genes encoding proteins with optimal characteristics for vaccine design (i.e., surface-exposed or secreted proteins, conservation among different strains, prediction of B-cell and T-cell immune epitopes) [46]. Selected candidates will be then expressed and used to immunize animal models for the study of the immunogenicity and protection against the pathogen infection. A more recent approach, termed “structural vaccinology”, involves analysis of the 3D structure of a selected antigen and vaccine testing of individual antigenic domains [47,48]. This approach has been shown as particularly useful when vaccine antigens are prone to antigenic variation. However, these novel approaches have limitations, such as the inability to identify nonprotein antigens, such as polysaccharides, that are components of some successful vaccines.

The design of subunit vaccines targeting Bcc presents several challenging difficulties, resulting from the ability of Bcc to form biofilms, to secrete proteases, to survive intracellularly, and to evade the immune system [49,50,51]. In addition, strain-specific virulence traits such as the differences in the strategies used by different Bcc species to survive intracellularly add further complexity to the development of an effective vaccine. To tackle this pathogen efficiently, the immune system needs to evoke both a humoral and a cellular response. The knowledge of Th1 (cell-mediated response) and Th2 (humoral immune response) responses required for pathogen clearance from the host is essential for vaccine development. However, limited information is currently available about the cellular and humoral response of the CF host to Bcc infection. CF patients have a partially compromised immune phenotype that appears to be unbalanced towards Th2 responses and with reduced Th1 responses [52]. However, a Th1 response has been reported to be important for the clearance of CF-related pathogens such as Bcc in some animal models [23,25,27]. Elicitation of a Th1 response may be advantageous to balance the Th1/Th2 response and produce a more efficient immune response in the Th2 CF lung. Routine national vaccines for the general population are recommended and currently performed for CF patients, and vaccination against influenza, hepatitis A and B, and Varicella Zoster virus is also recommended [53,54].

Adaptive immunity is mediated by T and B cells, which are capable of developing pathogen-specific memory, conferring immunological protection. These cells, through receptors, recognize specific parts of the antigens of the pathogen, known as epitopes. Epitopes have been shown to be protein regions that are flexible, surface-exposed, and usually hydrophilic, and normally corresponded to sites of turns and loops in folded proteins [55]. Epitopes are extensively employed in the development of immunodiagnostic tests, antibody-based therapeutics, and vaccines, allowing the replacement of complete antigens that are potentially pathogenic [56]. The development of multi-epitope vaccines against bacterial infections, based on the production of chimeric proteins by recombinant DNA technology, has been pursued by several research groups [57,58]. Multi-epitope vaccines present several advantages, such as the inclusion of various immunoprotective epitopes in a single molecule and the exclusion of immunodominant, but not immunoprotective, epitopes. However, the major drawback of this approach is the correct identification of the conserved immunoprotective epitopes. Epitope identification is expensive and laborious, requiring experimental screening of large arrays of potential epitope candidates. However, recent works of epitope mapping of *Burkholderia* strains have been performed using the peptide microarray approach [59,60,61]. This technique relies on the use of libraries of synthesized linear peptides spotted on a single chip and screening for antigenicity using patients’ antisera. 

Therefore, several bioinformatics tools were developed to aid in the selection of putative immunoprotective epitopes for both B and T cells. These tools are quite useful in predicting epitopes, minimizing costs and time-consuming experimental work. The identification of the antigenic epitopes is based on evolutionary conservation of surface residues, on physicochemical properties of the surface region (e.g., interface propensity, desolvation properties, or hydrophobicity), or on geometric properties (e.g., shape of the surface region or residue mobility) [62].

B-cell epitopes can be categorized as discontinuous (conformational) or continuous (linear) epitopes, although the majority of the epitopes are discontinuous [63]. In the case of discontinuous epitopes, amino acid residues are in close contact due to their three-dimensional conformation. The minimal contact of amino acid residue sequence required for proper folding of the discontinuous epitope in native proteins may range from 20 to 400 amino acid residues. The vast majority of discontinuous epitopes (over 70%) are composed of 1–5 linear segments of lengths of 1–6 amino acids [64]. Therefore, the identified linear epitopes can be part of the conformational B-cell epitope. Recently, using the Bepipred tool for identification of continuous B-cell epitopes, we identified in silico three putative outer membrane proteins with higher probability to be immunogenic and conserved in Bcc [24]. In particular, the BCAL2958 protein was experimentally demonstrated to be expressed by all the 12 analyzed strains from seven different Bcc species [24]. The presence of anti-BCAL2958 antibodies in sera from cystic fibrosis patients with a clinical record of respiratory infection by Bcc and the ability of the purified protein to stimulate neutrophils in vitro was also observed [24]. Structure-based epitope prediction was also recently performed to identify epitopes for the design of new diagnostic molecules for the early detection of *Burkholderia* infections [65]. A list of selected web tools for B-cell epitope prediction is presented in Table 1.

In contrast with B cells, that recognize most of the conformational epitopes from proteins, T cells recognize linear epitopes that are processed by antigen-presenting cells (APCs) [87]. Peptides binding with higher affinity to Major Histocompatibility Complex (MHC) have higher probability to be displayed on the cell surface and be recognized by a T-cell receptor [87]. The knowledge of these epitopes is important when designing subunit vaccines conferring cellular immunity. A list of selected web tools for T-cell epitope prediction is presented in Table 2.

## 5. Final Remarks and Perspectives

Despite the advances in the taxonomy and epidemiology of Bcc infections, as well as the availability of an increasing number of sequenced genomes of these bacteria, infections remain an important cause of morbidity and mortality among CF patients. In addition, several reports of Bcc infections among non-CF patients suggest the emergence of Bcc infections among patients hospitalized for prolonged periods of time and suffering from other malignancies. The development of a protective vaccine towards Bcc infections is quite attractive, but many obstacles remain to be solved. For instance, the fact that these bacteria can survive and replicate intracellularly requires the induction of both humoral and cellular immunological responses to effectively enable the host to cope with Bcc. Subunit vaccines and live attenuated vaccines appear to be the main focus of Bcc vaccine research. The currently available genomic information and bioinformatics tools greatly enhance our ability to identify immunogenic proteins which are potential candidates to develop subunit vaccines. It is worth to mention that despite these advances, the rational identification of immunogenic proteins and their ability to confer protection still needs to be experimentally confirmed. Other issues related to the development of a vaccine to protect against Bcc infections will certainly deserve future research attention, namely the partially compromised immune phenotype of the CF patient’s immune system and how to modulate it.

## Figures and Tables

**Table 1:** Selected web tools for in silico B-cell epitope prediction.

Tools	Link	Description	Reference
Pepitope	http://pepitope.tau.ac.il/	Prediction of linear and discontinuous B-cell epitopes using Pepsurf or Mapitope algorithm	[66]
Epitopia	http://epitopia.tau.ac.il/	Prediction of linear and discontinuous B-cell epitopes	[67]
Ellipro	http://tools.immuneepitope.org/ellipro/	Prediction of linear and discontinuous B-cell epitopes based on the protein antigen’s 3D structure	[68]
BepiPred 2.0	http://www.cbs.dtu.dk/services/BepiPred/	Prediction of linear B-cell epitopes	[69]
Bcepred	http://crdd.osdd.net/raghava/bcepred/	Prediction of linear B-cell epitopes using physicochemical properties	[70]
ABCPred	http://crdd.osdd.net/raghava/abcpred/	Prediction of linear B-cell epitopes using recurrent neural network	[71]
BEST	http://biomine.cs.vcu.edu/datasets/BEST/	Prediction of linear B-cell epitopes using support vector machine (SVM) tool	[72]
SVMTriP	http://sysbio.unl.edu/SVMTriP/prediction.php	Prediction of linear B-cell epitopes using SVM and combining tripeptide similarity and propensity scores	[73]
AAPPred	https://bioinf.ru/aappred/predict	Prediction of linear B-cell epitopes using amino acid pair antigenicity scale	[74]
COBEpro	http://scratch.proteomics.ics.uci.edu/	Prediction of linear B-cell epitopes	[75]
BCPREDS	http://ailab.ist.psu.edu/bcpred/predict.html	Prediction of linear B-cell epitopes using AAP, BCPred, or FBCPred method	[76,77,78]
LBtope	http://crdd.osdd.net/raghava/lbtope/protein.php	Prediction of linear B-cell epitopes	[79]
CBTOPE	http://crdd.osdd.net/raghava/cbtope/submit.php	Prediction of discontinuous B-cell epitopes	[80]
PEASE	http://www.ofranlab.org/PEASE	Prediction of discontinuous B-cell epitopes	[81]
BEpro	http://pepito.proteomics.ics.uci.edu/	Prediction of discontinuous B-cell epitopes	[82]
DiscoTope 2.0	http://www.cbs.dtu.dk/services/DiscoTope/	Prediction of discontinuous B-cell epitopes	[83]
EPCES	http://sysbio.unl.edu/EPCES/	Prediction of discontinuous B-cell epitopes	[84]
EpiPred	http://opig.stats.ox.ac.uk/webapps/sabdab-sabpred/EpiPred.php	Prediction of discontinuous B-cell epitopes	[85]
EPSVR	http://sysbio.unl.edu/EPSVR/	Prediction of discontinuous B-cell epitopes	[86]

**Table 2:** Selected web tools for in silico T-cell epitope prediction.

Tools	Link	Description ^a^	Reference
SYFPEITHI	http://www.syfpeithi.de/bin/MHCServer.dll/EpitopePrediction.htm	MHC I and MHC II binding prediction	[88]
MHCPred	http://www.ddg-pharmfac.net/mhcpred/MHCPred/	MHC I and MHC II binding prediction	[89]
RANKPEP	http://imed.med.ucm.es/Tools/rankpep.html	MHC I and MHC II binding prediction	[90]
SVMHC	https://abi.inf.uni-tuebingen.de/Services/SVMHC	MHC I and MHC II binding prediction	[91]
SVRMHC	http://c1.accurascience.com/SVRMHCdb/	MHC I and MHC II binding prediction	[92]
IEDB	http://tools.iedb.org/main/tcell/	MHC I and MHC II binding prediction	[93]
EpiJen	http://www.ddg-pharmfac.net/epijen/EpiJen/EpiJen.htm	MHC I binding prediction	[94]
nHLAPred	http://crdd.osdd.net/raghava/nhlapred/	MHC I binding prediction	[95]
ProPred 1	http://crdd.osdd.net/raghava/propred1/	MHC I binding prediction	[96]
NetMHC 4.0	http://www.cbs.dtu.dk/services/NetMHC/	MHC I binding prediction	[97]
PREDEP	http://margalit.huji.ac.il/Teppred/mhc-bind/	MHC I binding prediction	[98]
NetCTL 1.2	http://www.cbs.dtu.dk/services/NetCTL/	MHC I binding prediction	[99]
ProPred	http://crdd.osdd.net/raghava/propred/	MHC II binding prediction	[100]
MHC2Pred	http://crdd.osdd.net/raghava/mhc2pred/	MHC II binding prediction	[101]

^a^ Abbreviations: MHC I—Major Histocompatibility Complex I; MHC II—Major Histocompatibility Complex II.
